# Predictors of teenage pregnancy among girls aged 13–19 years in Uganda: a community based case-control study

**DOI:** 10.1186/s12884-019-2347-y

**Published:** 2019-06-24

**Authors:** Anthony Mark Ochen, Primus Che Chi, Stephen Lawoko

**Affiliations:** 1 School of Public Health, Makerere University, P. O. Box, Kampala, 7072 Uganda; 2 KEMRI-Wellcome Trust Research Programme, P.O .Box 230-80108, Kilifi, Kenya; 3 Department of Public Health Sciences, Karolinska Institutet, SE-171 77 Stockholm, Sweden; 4 Peace Research Institute Oslo (PRIO), Oslo, Norway; 5 Faculty of Health Sciences, Victoria University, P. O. Box, Kampala, 30886 Uganda

**Keywords:** Teenage pregnancy, Adolescent, Behavioural factors, Familial factors, And social factors

## Abstract

**Background:**

Teenage pregnancy is a serious public health and social problem, with 95% occurring in developing countries. The aim of the study was to investigate the behavioural, familial and social factors associated with teenage pregnancy among girls aged 13–19 years in Lira District, Uganda.

**Methods:**

Primary data from a case-control study of teenage girls (aged 13–19 years) in Lira District, Uganda was analysed. A Structured questionnaire was administered using face-to-face interviews to collect data on 495 participants, identified through simple random sampling from 32 villages in two counties in Lira District. Data analyses were done using SPSS Statistics 23 for descriptive, bivariate (i.e. Chi-square tests) and multivariable analyses (i.e. logistics regression) used for determining independent associations.

**Results:**

A total of 495 teenage girls participated in the study, however, final analyses were undertaken for 480 respondents. At bivariable analysis, all variables except alcohol consumption were significantly associated with teenage pregnancy. Among the behavioural factors assessed, multivariable analyses showed that having multiple sexual partners, frequent sex and irregular contraceptive use increased the likelihood of teenage pregnancy. Among familial factors, being married was found to increase the likelihood of teenage pregnancy. Peer pressure, sexual abuse and lack of control over sex was observed to increase the likelihood of teenage pregnancy.

**Conclusions:**

Demographic**,** behavioural, familial and social factors are important predictors of teenage pregnancy in Lira District. Interventions focussing on: retaining pregnant and married girls at school, information on sexual and reproductive health of teenage girls, improving access to and information about contraceptive use among teenage girls, improving socio-economic status of households, and law enforcement on sexual abuse among girls may come a long way to improving adolescent sexual and health services in the low-income settings.

**Electronic supplementary material:**

The online version of this article (10.1186/s12884-019-2347-y) contains supplementary material, which is available to authorized users.

## Background

The United Nations Children Fund (UNICEF), defines teenage pregnancy as “a teenage girl, usually within the ages of 13-19, becoming pregnant and refers to girls who have not reached legal adulthood, which varies across the world” [1]. Although it is considered a serious public health and social problem globally [2], approximately, 95% occur in developing countries [3]. Teenage girls aged 15–19 years are twice more likely to die during pregnancy and childbirth compared to women in their twenties, whereas those under the age of 15 years are five times more likely to die [4]. According to the World Health Organisation (WHO), most of the pregnancies and childbirth are not planned and wanted, although a few are planned and wanted [5]. Some of the complications associated with teenage pregnancy include: preterm labour, intrauterine growth retardation and low birth weight [6]; neonatal death, obstructed labour, genital fistula and eclampsia [7]. Furthermore, their reproductive health is affected by unsafe abortion, sexually transmitted infections, sexual violence and limited access to medical services [8].

The factors contributing to teenage pregnancy are multifactorial, ranging from individual-behaviour, traditional, and socio-cultural to religious in nature. Inarguably, low socio-economic status [9, 10], limited education [11], and early sexual activity [12] can perpetuate teenage pregnancy. Additionally, weak implementation of the Penal Code Act (which criminalizes sexual intercourse with girls below 18 years) and the Uganda National Adolescent Reproductive Health Policy by government institutions and a lack of community, social support and poverty are some of the determinants of teenage pregnancy. Furthermore, increased accessibility to social media and pornographic sharing [8], cross cultural influences, and decreased supervision by adults, have led to early initiation of sexual activity by teenagers [13]. Studies have shown substantial reduction in birth rates globally, with Adolescent Birth Rate (ABR) declining from 61.8 to 22.3% per 1000 female adolescents aged 15–19 years [14]. However, sub-Saharan Africa continue to have the highest ABR [15].

Uganda has one of the highest rates of teenage pregnancies in sub-Saharan Africa, estimated at about 25% [16]. Within the same country there are differences in the proportion with Lira District having the highest rate in Northern Uganda [17]. Socio-cultural and religious norms promote abstinence until marriage. However, as in many other societies, a double standard concerning sexuality is prevalent whereby girls are expected to be modest, tender, submissive and passive, while boys are encouraged to engage in behaviours that assert their masculinity, autonomy, and ambition [18].

The Uganda national adolescent reproductive health policy (2004) pledges commitment to advocate for the review of existing legal, medical and social barriers to adolescents’ access to information and health services. In addition to ensuring protection of the rights of adolescents to health, provision of legal and social protection against all forms of abuse and harmful traditional practices, promotion of gender equality and provision of quality care for adolescent sexual and reproductive health issues [19].

In spite of the implementation of available policies and other related laws, teenage pregnancies remain quite high in Uganda, especially in Lira District. This study therefore sought to investigate the behavioural, familial and social factors associated with pregnancy among teenage girls aged 13–19 years in Lira District, Uganda. The findings from this study will provide information that can be used by government institutions, health administrators and other relevant stakeholders to strengthen the implementation of the existing laws around national health policy, school health policy, national adolescent health policy and penal code act among others. Furthermore, it will provide policy makers with context-specific information for formulating policies that promote education, use of contraceptive methods and support sexual and reproductive rights of teenage girls.

## Methods

### Study context

This is a quantitative study using primary data from a case-control study of teenage girls conducted in Erute North County and Lira Municipal Council, Uganda. The aim of the study was to investigate behavioural, familial and social factors associated with pregnancy among teenage girls aged 13–19 years in Lira District, Uganda. Uganda is a low income country whose economy is predominantly agricultural, with majority of the population dependent on subsistence farming. It has a total population of 34.6 million persons, total fertility rate of 5.8 and life expectancy at birth for females and males is 64.2 and 62.2 respectively [20]. Lira District is divided into three counties including one urban area, 13 Sub-counties, 89 parishes and 751 villages. The study was, however, conducted in two counties, four sub-counties, eight parishes and 32 villages. The total population of the District is 408,043 people with approximately 12.5% of these being teenage girls aged 13–19 years [20].

### Study design and sampling strategy

This was a case-control study design using quantitative data analysis. The sample size of 495 participants was determined with cases (n) to controls (2n) ratio [21], using standard normal value of 1.96 and power of 80%, where *n* = $\frac{{\left[{Z}_{\propto /2}\sqrt{\left(1+1/c\right)\overline{pq}}+{Z}_{\beta}\sqrt{p_1{q}_1+\frac{p_0{q}_1}{C}}\right]}^2}{{\left({P}_1-{P}_0\right)}^2}$. Simple random sampling without replacement (using lottery method) was used to identify 32 villages in two counties. A list of teenage girls (sampling frame of teenage girls which was generated from the National Population and Housing Census of Uganda, 2014) was obtained from planning unit in the District and samples proportionately distributed among selected villages. Simple random sampling was further used to identify participants in the household with more than two eligible teenage girls.

### Measurements

The development of the questionnaire was informed by in-depth literature review and adaptation of related questions from the previous studies related to teenage pregnancy, (questionnaire in Additional file 2). The questionnaire was designed in English language and later translated into Luo language (the first language of respondents) and then translated back into English with the help of Lira District health educator and other experienced health workers in the district. The variables measured comprised of: demographic characteristics, behavioural, familial, and social factors as shown in Table 1. The variables used in the study were measured as follows (detailed explanation in Additional file 1).

**Table 1:** Measurement of variables

Main variables	Sub-variables	Measurements
Dependent variable	Currently pregnant	Yes/no
Demographic characteristics	Age of teenage girl	Age in completed years
	Place of residence	Rural/urban area
	School attendance	Still in school or not
	Parental educational level	No education, primary education, secondary education or post-secondary education
	Parental occupation	Farmer, business, government/non-governmental organisation or others…
	Parents alive	Father/mother alive or dead
Behavioural factors	Age at first sexual intercourse	Age in completed years
	Multiple sexual partners	Having concurrent number of sexual partners
	Frequency of sexual intercourse	Average number of sexual intercourse per week
	Contraceptive use	Regular use of contraceptive methods during sexual intercourse – yes/no
Familial factors	Household socio-economic status	Ownership of households properties, categorized as; high socio-economic status, medium socio-economic status or low socio-economic status
	Marital status	Currently married or not
	Parental divorce/separation	Father/mother divorced/separated
	Person teenage lived with	Father, mother, both parents, relative or husband
	Domestic violence	Yes/no
	Physical neglect	Yes/no
Social factors	Peer pressure	Yes/no
	Sexual abuse	Yes/no
	Control over sex	Yes/no
	Awareness on adolescent sexual and reproductive health	Yes/no
	Perception of cultural norm on sex before 18 years	Yes/no

### Recruitment of study participants

The study participants were teenage girls of age 13–19 years who were either cases (pregnant girls) at the time of the interview or controls (non-pregnant girls) at the time of the interview. All of them were recruited by six research assistants. Each day, they proceeded to the homes of Village Health Teams (VHTs) who guided them to the households where the study participants live. Cases were first identified by probing their pregnancy status followed by the controls, and for every case, two controls were identified either within the same households or neighbouring households. Sexual activity among controls was determined by asking: frequency of sex per week, number of concurrent sexual partners, and use of contraceptive methods during sex. The parents/guardians of participants would first be informed of the purpose of the study, then asked for permission to interview the girls. The confidentially of information was guaranteed by using codes instead of names. Furthermore, interview of respondents were conducted in places with no interferences from other people. The informed consent document was read to him/her and signed by both after agreement. Similarly, informed consent was sought from cases, however, controls were administered assent forms due to the fact that they were minors. Sexual activity among controls was determined by asking the frequency of sex per week, number of concurrent sexual partners, and use of contraceptive methods during sex. The households where either parents/guardians or teenage girls declined to participate were excluded and another household selected. The study excluded those who were too ill or did not consent to participate in the study. Data collection was conducted for a total of 10 days in the month of July 2016.

A total of 495 participants (165 cases and 330 controls) were recruited in the study but after thorough data checking, 15 questionnaires (5 for cases and 10 for controls) were excluded due to incomplete information. Final analyses were performed on 480 participants (160 cases and 320 controls).

### Data analysis

The SPSS Statistics 23.0 was used for analyses. Descriptive characteristics of participants were presented as frequencies and percentages. For bivariate analyses, cross tabulations were applied to study association between the predictor variables and the dependent variable. Associations were tested using Pearson Chi-Square (x^2^) tests. Statistical significance was obtained using 95% Confidence Interval (CI) at *p* < 0.05. Significant variables at *p* < 0.05 were further analysed at multivariable level. Multicollinearity check was performed using the variance inflation factor error cut-off of below seven.

Hierarchical logistic regression analysis was conducted to predict teenage pregnancy using behavioural, familial and social factors as potential predictor variables. These variables were entered into the model block-wise [22], with the blocks designated based on the theory. Several models were presented with Model 1 including only demographic variables; Model 2 demographic and behavioural factors; Model 3 demographic, behavioural and familial variables; and Model 4 demographic, behavioural, familial and social variables. Model robustness was assessed using − 2 Log Likelihoods, Nagelkerke pseudo R^2^ was used to compare the differences between models and goodness of fit using Hosmer and Lemeshow test. Effect modification was performed on some of the independent factors in order to assess the interaction effects on the outcome.

### Ethical issues

The study received ethics approval from the Higher Degrees, Research and Ethics Committee (HDREC), Makerere University School of Public Health, Kampala (Uganda). Further authorisation was granted by the District Health Officer (DHO) of Lira District. Informed consents were obtained from the cases/pregnant girls and parents/guardians of controls/non-pregnant girls below the age of 18 years and assent from the non-pregnant girls below the age of 18 years. Written consent was given by the pregnant girls, non-pregnant girls above 18 years and parents/guardians of non-pregnant girls below the age of 18 years prior to administration of the questionnaire to participants. Pregnant adolescent girls gave their individual written consents without that of their parents/guardians. The parental consent of pregnant adolescent girls was waived by the IRB due to the fact that they are emancipated minors and able to make their own decisions. However, written parental consent was given for non-pregnant girls below the legal age of 18 years. All the girls who had experienced sexual abuse and needed help were referred to nearby health facilities for counselling and treatment, and those neglected by their parents were referred to police children and protection unit. Phone contacts for principal investigators were given to them in case they needed help related to their situations under study.

## Results

### Respondent characteristics

Data on 480 teenage girls were analysed as presented in Table 2. About 60% of the respondents were living in urban areas, over 78% were between the ages of 15–19 years and 22% between the ages of 13–14 years, majority of the respondents (90.3%) had first sexual encounter in life at older age (15–19), 34.8% of them were married and about half were still attending school. Approximately, 50% of their fathers and 33% of their mothers had attained post-secondary level of education. Further characteristics showed that most of the parents were alive (72.3%) and were employed as farmers (27.7%), with a substantial proportion of the families within the low socio-economic class (40.6%). There was relatively high proportion (66.0%) of domestic violence among family members and physical neglect (57.3%), however, parental separation was less than a quarter of all the total samples in the study. Higher prevalence were also observed among participants who had peer pressure (56.2%) and lack of control over sex (54.2%) respectively, those who were sexually abused were about 34%.

**Table 2:** Descriptive characteristics of study respondents (*N* = 480), Lira (Uganda)

Variables	Characteristics	n (%)
1. Place of residence	Rural	195 (40.6)
	Urban	285 (59.4)
2. Age group (years)	Younger teenagers (13–14)	102 (21.3)
	Older teenagers (15–19)	378 (78.7)
3. School attendance	Yes	246 (48.8)
	No	234 (51.2)
4. Father’s education	No education	33 (6.9)
	Primary education	104 (21.7)
	Secondary education	106 (22.1)
	Post-secondary education	237 (49.4)
5. Mother’s education	No education	81 (16.9)
	Primary education	134 (27.9)
	Secondary education	105 (21.9)
	Post-secondary education	160 (33.3)
6. Parents alive	Yes	347 (72.3)
	No	133 (27.7)
7. Type of parents’ occupation	Farmer	157 (32.7)
	Business person	150 (31.3)
	Government/NGO employed	53 (11.0)
	Others*	120 (25.0)
8. Age at first sex (*n* = 360)	Younger age	35 (9.7)
	Older age	325 (90.3)
9. Marital status of teenage girls	Yes	167 (34.8)
	No	313 (65.2)
10. Parental separation/divorce	Yes	113 (23.5)
	No	367 (76.5)
11. Socio-economic status	High	133 (27.7)
	Medium	152 (31.7)
	Low	195 (40.6)
12. Domestic violence	Yes	317 (66.0)
	No	163 (34.0)
13. Physical neglect	Yes	275 (57.3)
	No	205 (42.7)
14. Peer pressure	Yes	270 (56.2)
	No	210 (43.8)
15. Sexual abuse	Yes	162 (33.8)
	No	318 (66.2)
16. Lack of control over sex (*n* = 360)	Yes	165 (45.8)
	No	195 (54.2)

*N* = number of participants, *n* = frequency of participants, % = percent, others* carpenters, builders and welders

### Bivariable analysis

All the demographic variables were significantly associated with teenage pregnancy at *p* < 0.001 as shown in Table 3. The prevalence of teenage pregnancy among older teenagers was higher than their younger peers, teenage girls living in rural areas had higher proportion of teenage pregnancy than those in urban areas, and the likelihood of teenage pregnancy was higher among non-school goers than those attending school. Additionally, the prevalence of teenage pregnancy was higher among girls whose parents were employed as peasant farmers than those employed by Government/NGO and businesses.

**Table 3:** Analysis of demographic, behavioural, familial and societal factors with teenage pregnancy

Variables	Pregnant girls *n* (%)	Test statistics X^2^ (df)	
Demographic variables			
Age group			
Older teenagers (15–19) (*N* = 378)	152 (40.2)	37.87*** (1)	
Younger teenagers (13–14) (*N* = 102)	8 (7.8)		
Place of residence			
Rural (*N* = 195)	82 (42.1)	11.23*** (1)	
Urban (*N* = 285)	78 (27.4)		
School attendance by teenage girls			
In school (*N* = 246)	8 (3.3)	205.48*** (1)	
Not in school (*N* = 234)	152 (65.0)		
Father’s education			
No education (*N* = 33)	21 (63.6)	16.54*** (3)	
Primary education (*N* = 104)	38 (36.5)		
Secondary education (*N* = 106)	30 (28.3)		
Post-secondary education (*N* = 237)	71 (30.0)		
Mother’s education			
No education (*N* = 81)	46 (56.8)	31.87*** (3)	
Primary education (*N* = 134)	44 (32.8)		
Secondary education (*N* = 105)	37 (35.2)		
Post-secondary education (*N* = 160)	33 (20.6)		
Type of parents’ occupation			
Farmer (*N* = 157)	46 (29.3)	19.68*** (3)	
Business (*N* = 150)	45 (30.0)		
Government/employed (*N* = 53)	32 (60.4)		
Others* (*N* = 120)	37 (30.8)		
Behavioural variables			
Age at first sex			
Younger age (13–14) (*N* = 35)	23 (65.7)	7.10** (1)	
Older age (15–19) (*N* = 325)	137 (42.2)		
Multiple sexual partners			
Yes (*N* = 111)	89 (80.2)	83.00*** (1)	
No (*N* = 249)	71 (28.5)		
Frequency of sex in a week			
Less than two times (*N* = 244)	70 (28.7)	76.13*** (1)	
More than two times (*N* = 116)	90 (77.6)		
Frequency of alcohol use per week			
Yes (*N* = 130)	52 (40.0)	2.21 (1)	
No (*N* = 56)	29 (51.8)		
Frequency of contraceptive use			
Never (*N* = 112)	20 (17.9)	46.55*** (1)	
Rarely (*N* = 248)	140 (56.5)		
Familial Variables			
Socio-economic status of families			
High (*N* = 133)	38 (28.6)	7.62* (2)	
Medium (*N* = 152)	43 (28.3)		
Low (*N* = 195)	79 (40.5)		
Marital status of teenage girls			
Married (*N* = 167)	86 (51.5)	38.02*** (1)	
Not married (*N* = 313)	74 (23.6)		
Parental separation/divorce			
Yes (*N* = 113)	54 (47.8)	13.90*** (1)	
No (*N* = 367)	106 (28.9)		
Person teenager is living with			
Live with both parents (*N* = 212)	54 (25.5)	21.43*** (4)	
Live with only father (*N* = 39)	11 (28.2)		
Live with only mother (*N* = 92)	44 (47.8)		
Live with husband (*N* = 66)	31 (47.0)		
Live with relative (*N* = 71)	20 (28.2)		
Domestic violence in families			
Yes (*N* = 317)	122 (38.5)	11.15*** (1)	
No (*N* = 163)	38 (23.3)		
Physical neglect of teenage girls			
Yes (*N* = 275)	108 (39.3)	10.22*** (1)	
No (*N* = 205)	126 (25.4)		
Parents alive			
Yes (*N* = 347)	98 (28.2)	14.61*** (1)	
No (*N* = 133)	62 (46.6)		
Societal Variables			
Peer pressure among teenagers			
Yes (*N* = 270)	116 (43.0)	25.75*** (1)	
No (*N* = 210)	44 (21.0)		
Sexual abuse of teenage girls			
Yes (*N* = 162)	97 (59.9)	77.53*** (1)	
No (*N* = 318)	63 (19.8)		
Control over sex among partners			
Yes (*N* = 165)	19 (11.5)	133.77*** (1)	
No (N = 195)	141 (72.3)		
Sexual and reproductive awareness			
Yes (*N* = 321)	78 (24.3)	35.59*** (1)	
No (*N* = 159)	82 (51.6)		
Perception of cultural norm on sex			
Yes allowed before 18 (*N* = 256)	106 (41.1)	16.09*** (1)	
Not allowed below 18 (*N* = 224)	54 (24.1)		

***p < 0.001, **p < 0.01, *p < 0.05, others* include carpenters, welders and builders, *x*^*2*^ chi square test, *df* degree of freedom, *N* = number of participants, and *n* = number of cases (pregnant girls)

The variables age at first sex, multiple sexual partners, frequency of sex and contraceptive use were all significantly associated with teenage pregnancy. Higher prevalence of teenage pregnancy was observed among younger teenagers who had experienced first sexual encounter in life, girls having multiple sexual partners, having sex more than twice a week, and those who rarely use contraceptive methods. The frequency of alcohol consumption was not significantly associated with teenage pregnancy, however, higher prevalence was observed among those who drink alcohol more than twice a week than those who drink alcohol less than twice a week.

All familial factors were significantly associated with teenage pregnancy. The prevalence of teenage pregnancy was higher among teenage girls whose families had low socio-economic status, girls who were married, and those whose parents had separated/divorced as compared with their counterparts who had no such occurrences. Higher prevalence was also reported among girls who experienced domestic violence and physical neglect.

All the social factors were significantly associated with teenage pregnancy at *p* < 0.001. Higher prevalence of teenage pregnancy was reported among girls who had intense peer pressure, had experienced sexual abuse and had no control over sex with partners than peers with no similar experiences. Similarly, the prevalence of teenage pregnancy was observed to be higher among girls who had no awareness on adolescent sexual and reproductive health than their counterparts who had such awareness. The likelihood of teenage pregnancy was higher among girls who reported that their culture allows sex before age 18 as compared to those who reported the contrary.

### Multivariable analysis

After adjusting for all other factors in the model 1 as shown in Table 4, the likelihood of teenage pregnancy among girls who were not attending school was significantly higher when compared with peers attending school (*p* < 0.001). Other variables such as: age of respondents, place of residence, parent’s education and occupation and whether parents were alive or not were not significantly associated with teenage pregnancy. However, after effect modification by marital status, age of respondents and place of residence became significantly associated with teenage pregnancy.

**Table 4:** The associations between teenage pregnancy and demographic, behavioural, familial and social factors, Lira (Uganda)

Variable	B	SE	Model 1 AOR (95%CI)	B	SE	Model 2 AOR (95%CI)	B	SE	Model 3 AOR (95%CI)	B	SE	Model 4 AOR (95%CI)
Demographic variables												
Age group (years)												
Younger teenagers			1			1			1			1
Older teenagers	0.32	0.80	1.4 (0.29–6.61)	−0.45	−1.14	0.7 (0.07–5.91)	− 0.54	1.23	0.6 (0.05–6.50)	0.34	2.05	1.4 (0.03–78.47)
Place of residence												
Urban			1			1			1			1
Rural	0.51	0.34	1.7 (0.86–3.23)	0.26	0.42	1.3 (0.58–2.93)	0.36	0.48	1.4 (0.55–3.68)	−1.20	0.82	0.3 (0.06–1.51)
School attendance												
In school			1			1			1			1
Not in school	3.83	0.44	45.9 (19.55–107.91)**	3.92	0.55	50.3 (17.25–146.96)**	3.84	0.65	46.6 (13.03–166.91)**	3.87	0.94	**47.7** (7.61–299.53)**
Fathers’ education												
Post-secondary education			1			1			1			1
No education	−0.06	0.55	0.9 (0.32–2.78)	0.28	0.74	1.3 (0.31–5.59)	0.16	0.84	1.2 (0.23–6.15)	1.00	1.17	2.7 (0.27–26.78)
Primary education	− 0.52	0.45	0.6 (0.25–1.44)	− 0.48	0.59	0.6 (0.20–1.97)	−0.29	0.71	0.7 (0.19–3.02)	1.30	1.22	3.7 (0.33–40.42)
Secondary education	−0.49	0.45	0.6 (0.26–1.47)	−0.34	0.55	0.7 (0.24–2.10)	−0.44	0.65	0.6 (0.18–2.30)	0.24	0.98	1.3 (0.19–8.66)
Mothers’ education												
Post-secondary education			1			1			1			1
No education	1.68	0.47	5.4 (2.15–13.35)**	1.77	0.62	5.8 (1.74–19.66)*	1.71	0.72	5.5 (1.35–22.67)*	0.47	1.09	1.6 (0.19–13.64)
Primary education	0.76	0.39	2.1 (0.98–4.61)	0.57	0.52	1.8 (0.64–4.88)	0.46	0.60	1.6 (0.49–5.18)	−0.40	0.80	0.7 (0.14–3.25)
Secondary education	1.18	0.45	3.2 (1.35–7.78)*	1.05	0.55	2.9 (0.97–8.44)	0.92	0.65	2.5 (0.71–8.97)	0.69	0.85	2.0 (0.38–10.63)
Type of occupation												
Business			1			1			1			1
Farmer	−1.11	0.48	0.3 (0.13–0.84)*	− 1.79	0.62	0.2 (0.05–0.57)*	−1.93	0.77	0.1 (0.03–0.66)*	−2.43	1.22	0.1 (0.01–1.95)
Employed (Government/NGO)	− 0.14	0.42	0.9 (0.39–1.97)	−0.38	0.50	0.7 (0.26–1.81)	−0.28	0.58	0.8 (0.25–2.35)	−0.35	0.85	0.7 (0.13–3.69)
(0.03–0.66)* (0.03–0.66)*Others*	−0.97	0.59	2.6 (0.83–8.45)	− 0.16	0.82	0.9 (0.17–4.27)	0.21	0.99	1.2 (0.18–8.56)	0.43	1.72	1.5 (0.05–44.77)
Parents alive												
Yes			1			1			1			1
No	0.06	0.31	1.1 (0.57–1.95)	.26	.39	1.3 (0.60–2.77)	0.19	0.58	1.2 (0.39–3.75)	0.78	0.84	2.2 (0.42–11.39)
Behavioural variables												
Age at first sex												
Older age						1			1			1
Younger age				1.66	0.78	5.2 (1.14–24.04)*	2.17	0.89	8.7 (1.53–49.69)*	1.84	1.34	6.3 (0.46–86.18)
Multiple sexual partner												
Yes						1			1			1
No				−2.49	0.47	0.1 (0.03–0.21)**	−3.05	0.59	0.5 (0.02–0.15)**	−3.49	0.83	**0.03** (0.01–0.16)***
Frequency of sex per week						1			1			1
More than two times				−1.79	0.45	0.2 (0.07–0.40)**	−2.06	0.55	0.1 (0.04–0.37)**	−1.95	0.76	**0.1** (0.03–0.63)**
Less than two times												
Contraceptive use												
Rare use						1			1			1
Often use				−1.86	0.46	0.2 (0.06–0.38)**	− 1.87	0.54	0.2 (0.05–0.44)**	−1.93	0.73	**0.1** (0.04–0.61)**
Familial Variables												
Socio-economic status												
High									1			1
Low							0.52	0.64	1.7 (0.48–5.96)	0.68	0.83	2.0 (0.39–10.05)
Medium							−0.74	0.70	0.5 (0.12–1.89)	−0.34	0.93	0.7 (0.12–4.36)
Marital status												
Married									1			1
Not married							−2.12	0.50	0.1 (0.05–0.32)**	−2.38	0.75	**0.1** (0.02–0.41)**
Parental separation/divorce												
Separated									1			1
Not separated							−0.56	0.52	0.6 (0.21–1.57)	−1.40	0.73	0.3 (0.06–1.03)
Person living with												
Live with relative									1			1
Live with both parents							−0.13	0.76	0.9 (0.20–3.91)	0.12	0.98	1.1 (0.17–7.78)
Live with only father							−0.24	0.91	0.8 (0.13–4.66)	−1.72	1.57	0.2 (0.01–3.87)
Live with only mother							0.65	0.74	1.9 (0.45–8.15)	1.04	0.97	2.8 (0.43–18.76)
Live with husband							0.41	0.80	1.5 (0.31–7.17)	1.26	1.13	3.5 (0.38–32.46)
Domestic violence												
Yes									1			1
No							−0.75	0.48	0.5 (0.18–1.22)	−1.13	0.71	0.3 (0.08–1.29)
Physical neglect												
Yes									1			1
No							−0.37	0.45	0.7 (0.29–1.69)	−0.56	0.66	0.6 (0.16–2.09)
Social Variables												
Peer pressure												
Yes												1
No										−1.95	0.76	**0.1** (0.04–0.69)**
Sexual abuse												
Yes												1
No										−1.79	0.72	**0.2** (0.04–0.69)**
Control over sex												
Yes												1
No										4.04	0.81	**57.0** (11.74–276.99)***
Awareness on ASRH												
Yes												1
No										0.70	0.70	2.0 (0.51–7.91)
Perception of culture												
Sex allowed before age 18												1
Sex not allowed before age 18										−0.67	0.64	0.5 (0.15–1.77)
Effect modification by marital status												
Age group												
Older teenagers*marital status										−5.28	0.82	**0.01** (0.01–0.03)***
Age at first sex												
Younger teenagers*marital status										−0.246	0.70	0.78 (0.20–3.07)
Place of residence												
Rural*marital status										0.77	0.29	**2.17** (1.23–3.82)**
School attendance												
Not in school*marital status										3.93	0.67	**50.98** (13.86–187.55)**
Control over sex												
No control*marital status										3.08	0.55	**21.81** (7.46–63.76)***
Socio-economic status												
Low*marital status										0.41	0.57	**1.51** (0.32–0.42)***
Medium*marital status										0.66	0.12	0.12 (0.49–4.59)
Domestic violence												
No violence*marital status										−0.79	0.31	**0.45** (0.25–0.83)**
Physical neglect												
No neglect*marital status										−0.98	0.30	**0.38** (0.21–0.67)***
Parental divorce												
No divorce*marital status										−0.93	0.28	**0.40** (0.23–0.69)***
Awareness												
No awareness*marital status										1.51	0.32	**4.52** (2.43–8.43)***
-2Log Likelihood			297.4			146.0			36.6			46.8
Hosmer & Lemeshow test			*P* = 0.35			*P* = 0.24			*P* = 0.49			*P* = 0.82
Nagelkereke pseudo R^2^			56.5%			19.0%			6.0%			27.2%
Classification accuracy			82.2%			92.2%			95.6%			96.9%

1 = reference category, ***p < 0.001, **p < 0.01, *p < 0.05, *AOR* adjusted odds ratio, *CI* confidence interval, *B* regression coefficient, *SE* standard error, others* = builders, carpenters and welders, *ASRH* adolescent sexual and reproductive health

Adjustment of behavioural factors in model 2 showed a few significant association of independent factors with teenage pregnancy. Age of teenage girls was found to be statistically insignificant with teenage pregnancy but after effect modification, older teenagers (15–19) were less likely to become pregnant as compared to younger ones. Multiple partners (*p* < 0.001), frequency of sex (*p* < 0.01) and contraceptive use (*p* < 0.01) significantly increased the likelihood of teenage pregnancy. Age at first sex was not significantly associated with teenage pregnancy when other factors were adjusted for in the logistic regression. Similarly, after effect modification by marital status, age at first sex still remained statistically insignificant.

Model 3 showed that only marital status remained significantly associated with teenage pregnancy (*p* < 0.01). Teenage girls who were not married were less likely to become pregnant as compared to those who were married. Others factors such as: socio-economic status, domestic violence, physical neglect, person the teenager is living with and parent’s separation/divorce were all not significantly associated with teenage pregnancy in the multivariable analyses. On the other hand, effect modification by marital status showed significant association with socio-economic status, domestic violence, parental divorce, and physical neglect.

In model 4, independent factors that remained significantly associated with teenage pregnancy include: peer pressure and sexual abuse. Experience of intense peer pressure (*p* < 0.01), sexual abuse (*p* < 0.01), and poor control over sex (*p* < 0.001), increased the likelihood of teenage pregnancy. On the other hand, cultural perception on sex and awareness on adolescent sexual and reproductive health were not significantly associated with teenage pregnancy. Effect modification however, showed significant association of awareness with teenage pregnancy after interaction by marital status.

Generally, there was improvement in the models after adjusting with all other factors. A test of the full model against a constant model was statistically significant, indicating that the predictors reliably distinguished between cases and controls.

## Discussions

This study described the behavioural, familial and social factors associated with pregnancy among teenage girls aged 13–19 years in Lira District, Uganda. At bivariate analyses, all variables except alcohol consumption were significantly associated with teenage pregnancy. At multivariable analyses: age of respondents, place of residence, school attendance, multiple sexual partners, frequent of sex, contraceptive use, socio-economic status, domestic violence, physical neglect, parental divorce/separation, peer pressure, sexual abuse, control over sex and awareness on adolescent sexual and reproductive health were found to be significantly associated with teenage pregnancy.

In the paragraphs that follow, a discussion of the key findings is presented with respect to the sub-topics: demographic, behavioural, familial, and social factors.

### Demographic factors

The results showed that age of the respondents and place of residence of respondents were not significantly associated with teenage pregnancy after adjusting for all independent factors. However, after effect modification by marital status, older teenagers (15–19) were found to be less likely at risk of teenage pregnancy as compared to younger teenagers (13–14). Teenage girls who resides in rural areas were twice more likely to become pregnant. On the other hand, being in school was found to be protective against teenage pregnancy. These findings are consistent with the previous studies in Uganda [16], Ethiopia [23] and Nepal [24]. Being young and living in rural areas may expose girls to early pregnancy due to: lack of information, peer influence and sexual abuse. This situation could put them in a higher risk of not only becoming pregnant but contracting sexually transmitted infections (STI). However, being in school may provide periods of supervision of teenage girls by teachers as well as parents, which could reduce opportunities for sexual activity [25].

### Behavioural factors and teenage pregnancy

The multivariable analysis on behavioural factors and teenage pregnancy shows that multiple sexual partners, frequency of sex, and contraceptive use were significantly associated with teenage pregnancy. Not having multiple sexual partners, having sex less than twice a week and regular use of contraceptive methods were all protective against teenage pregnancy. These results concur with a national study conducted in Uganda by the Uganda Bureau of Statistics (UBOS) in 2011 [26]. Another form of risky behaviour that result into teenage pregnancy have been found to be irregular use of contraceptive methods [13, 27–29], which is in agreement with our finding. As confirmed by this study, having multiple sexual partners puts teenage girls at greater risk of pregnancy [30]. Although this study did not address reasons for irregular contraceptive use, some of the contributory factors may be inadequate access, stigma and limited information on availability of contraceptive methods.

### Familial factors and teenage pregnancy

Multivariable analysis of familial factors and teenage pregnancy found a significant association only with marital status after adjustment with all other factors. However, at bivariate analysis, all familial factors were significantly associated with teenage pregnancy. However, after effect modification by marital status, socio-economic status, domestic violence, physical neglect, and parental divorce were found to be significantly associated with teenage pregnancy. These changes in the results are due to effect modification by marital status. Thus, marital status in this study should be taken as an effect modifier other than an independent predictor. Other studies found predominant association of early marriages (marriage of young girls) with teenage pregnancy [29, 31], which is consistent with this results. Low socio-economic status, and cultural traditions, especially payment of dowry as a source of income is most likely the issue exacerbating early marriages in Uganda. Economic deprivation is likely to influence teenage behaviours and heighten their exposure to early pregnancy as observed in Uganda [26], Nigeria [32], Sri Lanka [33], Senegal and Bangladesh [34], and Nepal [28]. Furthermore, there is growing concern that physical neglect of teenage girls could foster relationships with older men which is seen as more beneficial when daily needs such as food, shelter, clothing and money are not met by parents/caregivers [35].

### Social factors and teenage pregnancy

The results of multivariable analysis on social factors and teenage pregnancy reveals that peer pressure, sexual abuse, lack of control over sex and lack of awareness were significantly associated with teenage pregnancy. These results concur with some studies that have postulated that sexual abuse place girls at higher risk of experiencing teenage pregnancy [35–37]. Whereas, some researchers attribute the link between sexual abuse and teenage pregnancy to the adolescents’ behaviours [37], others maintain that existing evidence is still not conclusive [38]. Research supports the widespread idea that peers play an important role in teenage lives; teenagers with sexually active friends are more likely to have sex themselves [30]. Peers can influence the views of their age groups, hence, bad influence leading to risky behaviours such as: alcohol and drug abuse, dropping out of school, unprotected sexual activity which may lead to pregnancy [39]. This study concur with this analogy, as those who were not sexually abused were less likely to become pregnant. Community awareness on adolescent sexual and reproductive health was found to be significantly associated with teenage pregnancy after effect modification by marital status. This finding is similar to other studies that have demonstrated awareness creation as effective in reducing teenage pregnancy [11, 40, 41]. Furthermore, a survey of countries to assess their progress in implementation of the 1994 International Conference on Population and Development (ICPD) confirms that higher literacy rates among women between ages 15–19 was significantly associated with lower teenage birth rates [42].

### Implications for policy and programmes

This study provides useful findings that can be used to formulate policies and programmes towards addressing teenage pregnancy. The current study showed that teenage pregnancy is associated with teenage behaviours being perpetuated by familial and social factors. The current laws of Uganda; Penal Code Act (2007), which criminalizes sex with girls below 18 years (capital offense - punishable by death sentence) and National Adolescent Reproductive Health Policy (2004) are no longer current and are not fully operationalized by government institutions and society at large. The Uganda National Development Plan (NDP 2010–2014) acknowledges child marriage as a negative social cultural practice that increases the rate of early pregnancy, which is partly responsible for the persistent poor health outcomes for children and women especially high maternal and infant mortality rates and high fertility [43]. The Uganda government need to review existing legal, medical and social barriers to adolescent access to health information and reproductive health services, and further protect the rights of girls against all forms of abuse and harmful traditional practices. Provision of specific programmes that allow contraceptive use among teenage girls (from 13 to 19 years) in communities, sex education so that teenage girls avoid early sexual encounters and multiple sexual partners. The government should make necessary efforts to accommodate married and pregnant girls in schools. However, fresh philosophy on the effects of education on well-being also postulates that education alone is not enough to achieve successful transitions from adolescent into adulthood, and that girls need critical thinking skills as well as an enabling environment such as family and societal commitment and capacity for educating girls [44].

Due to some methodological limitations in the study, caution should be taken when generalizing these findings. However, it can be applied to other areas with low-income settings.

### Strengths and limitations of the study

The case-control design was used due to the fact that it is suitable when comparing two study groups (in this study cases and controls) and when exploring multiple exposures with one single outcome (teenage pregnancy). Besides, controls were drawn from the same population as cases thus minimising potential biases from both groups. Furthermore, simple random sampling technique allows even distribution of confounders among study participants. Adjusting for all other factors and assessing for effect modification helped to further reduce potential biases. Therefore, strengthening the association between predictor variables and the outcome. Lastly, the large sample size of 480 participants could have increased the power of the study as well.

The study has several limitations which are worth mentioning: only quantitative data was available for this study and yet it would have provided concretised findings had there been qualitative data; case-control design is prone to recall bias as participants have to recall some events that occurred sometimes in their lives and selection bias due to the fact that some girls selected as controls may have in fact been cases because of lack of disclosure caused by stigma surrounding teenage pregnancy. The results for school attendance and control over sex showed high point estimates with wide confidence intervals which could have reduced the level of precisions of their measures. Furthermore; pregnancy test was not conducted to confirm the pregnancy status of teenage girls, those who were one or 2 weeks pregnant could have not realized they were pregnant and therefore included as controls (non-pregnant).

## Conclusions

In conclusion, the study considered predictor (behavioural, familial and social) variables which were used to determine associations with teenage pregnancy. After adjusting with all other predictor variables and effect modification with marital status, demographic factors that became significantly associated with teenage pregnancy were: older age of respondents (15–19 years), living in rural areas and school attendance. Behavioural factors associated with teenage pregnancy in Lira District included: irregular contraceptive use, having multiple partners and frequent sex by teens. Familial factors significantly associated with teenage pregnancy were: being in a household with low socio-economic status, domestic violence, physical neglect and parental separation/divorce. Marital status was found to be an effect modifier other than independent predictor. Meanwhile social factors comprised of: peer pressure, sexual abuse, lack of control over sex and lack of awareness on adolescent sexual and reproductive health. The findings of this study can help to improve adolescent sexual and health services in low-income settings.

We therefore, recommend government to formulate programmes and policies aimed at: retaining married and pregnant girls in schools; promoting sex education aimed at abstinence from sex; allowing contraceptive use among teenage girls in communities and schools and ensure availability and accessibility of modern contraceptive methods; creating dialogue with parents with the view of discouraging early marriages of teenage girls; community sensitization so as to avoid groups that influence peers to engage in risky behaviours and early sex; strengthening the implementation of existing laws in order to deter sexual abusers; and promoting sexual and reproductive rights of teenage girls so that they have full control in making decisions concerning their sexual life. Finally, we suggest a more comprehensive study involving both quantitative and qualitative method for better understanding of how contextual factors influence teenage pregnancy.

## Additional files

Additional file 1: Explanation of variable measurements. (DOCX 15 kb)

Additional file 2: Structured Questionnaire of factors associated with teenage pregnancy among girls aged 13-19 years. (DOCX 20 kb)

## Data Availability

Not available. The study used secondary data from a larger study which is still being analysed, so release of data is restricted.

## Abbreviations

ABR: Adolescent Birth Rate
DHO: District Health Officer
GDP: Gross Domestic Product
HDREC: Higher Degrees, Research and Ethics Committee
STI: Sexually Transmitted Infection
UDHS: Uganda Demographic and Health Survey
UNICEF: United Nations Children’s Fund
VHTs: Village Health Teams
WHO: World Health Organisation
