# Supplementary material for: Predictors of teenage pregnancy among girls aged 13–19 years in Uganda: a community based case-control study
Source: BMC Pregnancy Childbirth. 2019 Jun 24;19:211. doi: 10.1186/s12884-019-2347-y (PMC6591948; doi:10.1186/s12884-019-2347-y)
Supplement: Supplementary file 1 — Explanation of variable measurements. (DOCX 15 kb) [file 12884_2019_2347_MOESM1_ESM.docx]

**Additional file 1 - Explanation of variable measurements**

**Dependent variable:**

The dependent variable was currently pregnant teenage girl. This was measured by asking the teenage girl whether she was pregnant or not. Probing questions were further used to determine whether she was actually pregnant or not.

**Demographic characteristics:**

Age of teenage girl: this was determined by asking the teenage girl her completed age in years, where she was not sure a school report card or immunization card was used to determine her age.

Place of residence: this was determined using geographical location where the teenage girl lived. It was measured as living in a rural or urban area.

School attendance: this was determined by asking the teenage girl whether she was still in school or dropped out of school. It was either a yes or no response.

Parental education: the teenage girl was asked whether her parents (father/mother) attained any educational standard. It was measured as; no education, primary level of education, secondary level of education or post-secondary level of education.

Parental occupation: the teenage girl was asked whether her father/mother was employed as; farmer, business person, government/non-governmental organisation or other employments….

Parents alive/dead: determined by asking the teenage girl whether her father/mother was alive or dead.

**Behavioural factors:**

Age at first sexual intercourse: the teenage girl was asked about her age in completed years at first sexual encounter in life. Probes were used where she was not sure.

Multiple sexual partners: this was determined by asking the teenage girl about having concurrent number of sexual partners at one time. Numbers were used to determine multiple sexual partners.

Frequency of sexual intercourse: determined by asking the teenage girl about the average number of sexual intercourse she had per week.

Contraceptive use: determined by asking whether the teenage girl regularly use any contraceptive methods when having sexual intercourse.

**Familial factors:**

Household socio-economic status: determined by using proxy indicators where the teenage girl was asked whether her household had possession of; permanent building, electricity, solar power, vehicle, motorcycle, television, bicycle, radio and animals. It was then categorized according to the value of the properties as high socio-economic status, medium socio-economic status and low socio-economic status.

Marital status: the teenage girl was asked whether she was currently married or not.

Parental divorce/separation: the teenage girl was asked whether her parents had divorced/separated.

Domestic violence: the teenage girl was asked whether her parents, step-parents, siblings, or any adult living with her pushed, grabbed, kicked, hit her with fist, threaten to hurt her with a knife or other tools, slapped or throw something at her most times. Any one of these responses was taken as domestic violence and no domestic violence if any was not mentioned.

Physical neglect: the teenage girl was asked whether she was not given enough food to eat at home, had torn clothes, had no sanitary pads when menstruating, had no money to buy breakfast and meals while at school, when sick had no one to take her for treatment, parents/caretaker always drink too much alcohol and unable to cater for her basic needs, no one to protect and take care of her needs most times. Any one of these responses was taken as physical neglect and no physical neglect if any was not mentioned.

**Social factors:**

Peer pressure: the teenage girl was asked whether she was pressured by friends to do what she didn’t want. It was measured as; never, often, quite often, and very often and further collapsed to no for never and yes for often/quite often/very often.

Sexual abuse: the teenage girl was asked whether any adult, relative, family friends or stranger older than her ever touched or fondled her body in a sexual way, made her touch their body in a sexual way, attempted to have any sexual encounter with her, and actually forced her to have sexual intercourse. Any one of these responses was taken as sexual abuse and no sexual abuse if any of these responses was not mentioned.

Control over sexual intercourse: the teenage girl was asked whether she had equal say over sexual intercourse as compared to her partner as regards when to have sexual intercourse or not, whether to use any contraceptive methods or not. It was either yes/no response.

Awareness on adolescent sexual and reproductive health: the teenage girl was asked whether her communities have her been sensitised on adolescent sexual and reproductive health, had information through reading newspapers, listening to radio, or watching television. The response was either a yes/no.

Perception of cultural norm as regards sexual intercourse of teenage girl below 18 years: the teenage girl was asked whether her cultural norm allows sexual intercourse with girls below the age of 18 years. The response was either a yes/no.
