# Supplementary material for: Predictors of teenage pregnancy among girls aged 13–19 years in Uganda: a community based case-control study
Source: BMC Pregnancy Childbirth. 2019 Jun 24;19:211. doi: 10.1186/s12884-019-2347-y (PMC6591948; doi:10.1186/s12884-019-2347-y)
Supplement: Supplementary file 2 — Structured Questionnaire of factors associated with teenage pregnancy among girls aged 13-19 years. (DOCX 20 kb) [file 12884_2019_2347_MOESM2_ESM.docx]

Additional file 2: Structured Questionnaire of factors associated with **teenage pregnancy among girls aged 13-19 years**

**Notes to data collector:**

**A case** is a female teenager of age group (13-19 years) who is pregnant at the time of the interview.

**A Control** is a female teenager of age group (13-19 years) who has never been pregnant at the time of interview.

ID Number: ……… Date: …/…./2016

Village…………………Parish: ……………………Sub-county: ………………………..

**A: Background characteristics**

1. Residence: a) Rural b) Urban
2. What is your age (in completed years)? ……
3. What is the level of your education?
4. No education b. primary education

c. secondary education d. post-secondary education

4. Are you still in school?

a. Yes b. No

1. Marital status:
2. Married b. Not married
3. What is your religion? a. Catholic b. Protestant c. Islam
4. Anglican e. Others, specify……………
5. Are your parents alive?
   1. Yes both of them are alive b. Yes only the father is alive

c. Yes only the mother is alive d. None of them is alive

8. Do you live with your parents/relatives?

a. I live with both parents b. I live with only the father

c. I live with only the mother d. I live with relative

**B: Family factors for teenage pregnancy**

1. What is the level of education of your father?
2. No education b. Primary education

c. Secondary education d. Tertiary education

1. What is the level of education of your mother?
   1. No education b. Primary education

c. Secondary education d. Tertiary education

1. What is the occupation of your parents/guardian?
2. Farmer b. Business person c. Government/NGO employee

d. carpenter/welder e. Others, specify………………..

1. Do you have the following items at home (tick all that apply)?
2. Permanent building b. Electricity c. Vehicle
3. Vehicle e. Motorcycle f. Bicycle
4. Television h. Radio i. Animals (cattle, goats, sheep) j. Monthly cash income k. Solar
5. Has any of your family member ever experienced teenage pregnancy?
6. Yes b. No

**C: Individual factors for teenage pregnancy**

1. Have you ever had sexual intercourse? ( if No skip to question 21)
2. Yes b. No
3. At what age did you have first sexual intercourse? .....................
4. Are you currently pregnant?
5. Yes b. No
6. How many times do you have sexual intercourse, say in a month?
   1. One time b. Two times

c. Three times d. More than three times

1. Have you ever had sexual intercourse with more than one partner?
2. Yes b. No
3. If yes, how often do you use contraceptives when having sexual intercourse?
4. Always b. Sometimes
5. Have you ever had any sexual education on the use of contraceptive methods?
6. Yes b. No
7. Have you ever been involved in alcohol consumption?
8. Yes b. No
9. If yes, how often do you drink, say in a week?
10. One time b, two times c. Three times

d. Four times e. every day f. Occasionally when at functions

**E: Socio-cultural factors for teenage pregnancy**

1. Do you feel any pressure from friends to do things you don’t want to?
2. Very often b. Never

c. Quite often d. Rarely often

1. Is it acceptable for somebody to have sexual intercourse before they are 18 years?
2. I strongly agree b. I strongly disagree

c. somewhat I agree d. somewhat I disagree

1. Is it culturally acceptable for a girl to get marriage at the age below 18 years?
2. I strongly agree b. I strongly disagree

c. somewhat I agree d. somewhat I disagree

1. Did you ever live with anyone who has a problem drinker or alcoholic?
2. Yes b. No
3. Did your parent, stepparent or adult living in your home push, grab, slap or throw something at you?
4. Not a victim of physical abuse
5. Victim of physical abuse at home

c. Victim of physical abuse in a foster home

d. physically abused by family member

e. Physically abused by someone outside the family

f. Attacked with a weapon

1. Has any adult, relative, family friend or stranger older than you ever;
2. Touched or fondled your body in asexual way?
3. Made you to touch his body in a sexual way?
4. Attempted to have any type of sexual intercourse (oral, anal or vaginal) with you?
5. Actually had any type of sexual intercourse (oral, anal or vaginal) with you?
6. Have you ever been faced with any of these situations at home?
7. Didn’t have enough food to eat, Yes No
8. There was someone there to take care of me and protect me Yes No
9. My parents were too drunk or too high to take care of me Yes No
10. I had to wear dirty clothes or torn clothes Yes No
11. There was someone to take me to the clinic/health unit if I needed it Yes No
12. Have you ever experienced any level of conflict or violence between parents, between youth and parents, among siblings?
13. Sometimes, often or very often pushed, grabbed, slapped or had something thrown at you
14. Sometimes, often or very often kicked, bitten, hit with a fist or hit with something hard
15. Ever repeatedly hit over at least a few minutes
16. Ever threatened with or hurt by a knife or other tools

**Thank you for your time!**
